# Supplementary material for: A systematic review of in vivo stretching regimens on inflammation and its relevance to translational yoga research
Source: PLoS One. 2022 Jun 1;17(6):e0269300. doi: 10.1371/journal.pone.0269300 (PMC9159623; doi:10.1371/journal.pone.0269300)
Supplement: S1 Table — (DOCX) [file pone.0269300.s001.docx]

**S1 Table. Electronic search strategy**

| **Searcher name, database, and date of searching** | **Syntax** | **Number of articles** |
| --- | --- | --- |
| Dennis Muñoz Vergara.  PubMed  2020.  Adding terms mobilization, manual therapy, and massage, eccentric muscle contraction, delated onset muscle soreness | (((Inflammat*[tiab] OR "Inflammation"[Mesh] OR "Inflammation Mediators"[Mesh] OR "Neurogenic Inflammation"[Mesh]) AND (immune*[tiab] OR immunology[tiab] OR inflammatory response*[tiab] OR cytokine*[tiab] OR interleukin*[tiab] OR resolving*[tiab] OR cortisol[tiab] OR vasodilat*[tiab] OR granulocyte*[tiab] OR opsonization[tiab] OR chemotaxis[tiab] OR agglutination[tiab] OR Lysosome granules*[tiab] OR histimine*[tiab] OR IFN-y[tiab] OR IL-8[tiab] OR Leukotriene B4[tiab] OR LTC4[tiab] OR LTD4[tiab] OR 5-oxo-eicosatetraenoic acid[tiab] OR 5-HETE[tiab] OR prostaglandin*[tiab] OR nitric oxide[tiab] OR cytokine*[tiab] tryptase[tiab] OR C-reactive protein[tiab] OR CRP[tiab] OR erythrocyte sedimentation[tiab] OR ESR[tiab] OR procalcitonin[tiab] OR PCT[tiab] OR serum amyloid A[tiab] OR alpha-1-acid glycoprotein[tiab] OR plasma viscosity[tiab] OR ceruloplasmin[tiab] OR hepcidin[tiab] OR haptoglobin[tiab] OR Inflammation resolution[tiab] OR Lipid mediator*[tiab] OR Resolvin*[tiab] OR pro-resolving mediator*[tiab] OR Eicosapentaenoic Acid[tiab] OR Docosahexaenoic Acid[tiab] OR Arachidonic acid[tiab] OR chemokine*[tiab])) AND ("Muscle Stretching Exercises"[Mesh] OR stretch*[tiab] OR flexible[tiab] OR flexibility[tiab] OR mechanotransduction[tiab] OR piezoelectric system[tiab] OR ((physical[tiab] OR active[tiab] OR passive[tiab] OR static[tiab] OR body[tiab] OR exercis*[tiab]) AND stretch*[tiab]) OR stretching exercise[tiab] OR ((muscular[tiab] OR muscle[tiab] OR connective tissue[tiab] OR fascia[tiab]) AND (extension OR extend[tiab] lengthen* [tiab] OR elastic*[tiab] OR flexibility[tiab] OR flex[tiab] OR stretch*[tiab] AND (mobilization*[tiab] OR manual therapy*[tiab] OR massage* [tiab] OR eccentric muscle contraction* [tiab] OR delayed onset muscle soreness*[tiab] OR DOMS*[tiab])))))  Filters: full text; other animals; English | **146** |
| Dennis Muñoz Vergara. EMBASE  2020.  Adding terms mobilization, manual therapy, and massage, eccentric muscle contraction, delated onset muscle soreness | ('rat'/exp OR 'mouse'/exp OR 'murine'/exp OR 'in vivo study'/mj OR 'muscle'/de OR 'connective tissue'/de OR 'connection tissue' OR 'connective sheath' OR 'connective tissue' OR 'subcutaneous tissue'/de OR 'hypodermis' OR 'subcutaneous tissue' OR 'subcutis' OR 'tela subcutanea' OR 'murine'/de OR 'murinae' OR 'murin' OR 'murine' OR 'murines') AND ('stretching'/exp OR 'stretching' OR 'mechanotransduction'/exp OR 'mechanical stretch'/exp OR 'cyclic mechanical stretch'/exp OR 'stretch-shortening contraction' OR 'muscle stretching'/de OR 'muscle stretch' OR 'muscle stretching' OR 'muscular stretch' OR 'muscular stretching' OR 'myotasis' OR 'stretch, muscle' OR 'stretching, muscle' OR 'stretching-shortening cycle' OR 'stretching exercise'/de OR 'muscle stretching exercises' OR 'stretching exercise' OR 'stretching exercises' OR 'muscle strain'/de OR 'muscle strain' OR 'muscle strains' OR 'strained muscle' OR 'stretch injured' OR 'dynamic force spectroscopy'/de OR 'stretch injury' OR 'passive stretch' OR 'active stretch' OR 'cumulative trauma disorder'/de OR 'cumulative trauma disorder' OR 'cumulative trauma disorders' OR 'injury, repetition strain' OR 'occupational overuse syndrome' OR 'overuse syndrome' OR 'repetition strain injury' OR 'repetitive motion disorder' OR 'repetitive strain injury' OR 'strain injury, repetition' OR 'repetitive stretch' OR 'mobilization'/exp OR 'manual therapy'/exp OR 'massage'/exp OR 'eccentric muscle contraction'/exp OR 'delayed onset muscle soreness'/exp) AND 'controlled study'/de AND ('inflammation'/exp OR 'acute inflammation' OR 'bacterial inflammation' OR 'inflammation' OR 'inflammation reaction' OR 'inflammation response' OR 'inflammatory condition' OR 'inflammatory lesion' OR 'inflammatory process' OR 'inflammatory reaction' OR 'inflammatory response' OR 'inflammatory syndrome' OR 'reaction, inflammation' OR 'response, inflammatory' OR 'serositis' OR 'sterile inflammation' OR 'inflammatory mediator'/exp OR 'specialized proresolving mediator'/de OR 'inflammation resolution') AND ('animal experiment'/de OR 'animal model'/de OR 'experimental model'/de OR 'in vivo study'/de OR 'mouse model'/de OR 'murine model'/de OR 'nonhuman'/de OR 'rat model'/de) AND ('article'/it OR 'article in press'/it) AND ('inflammation'/dm OR 'muscle injury'/dm OR 'muscle strain'/dm OR 'myofibrosis'/dm OR 'myositis'/dm OR 'paw edema'/dm OR 'wound healing'/dm) | **160** |
| Dennis Muñoz Vergara.  Web of Science  2020.  Adding terms mobilization, manual therapy, and massage, eccentric muscle contraction, delated onset muscle soreness | **TOPIC:** (rat*  OR mouse*  OR mus (genus)*  OR rattus (genus)*  OR in vivo study  OR murine*  OR murinae*  OR murin*  OR murine*  OR murines*) *AND* **TOPIC:** (muscle*  OR connective tissue*  OR connection tissue*  OR connective sheath*  OR connective tissue*  OR subcutaneous tissue*  OR hypodermis*  OR subcutaneous tissue*  OR subcutis*  OR tela subcutanea*) *AND* **TOPIC:** (stretching*  OR mechanotransduction*  OR mechanical stretch*  OR cyclic mechanical stretch*  OR stretch-shortening contraction*  OR muscle stretching*  OR muscle stretch* OR muscular stretch*  OR muscular stretching*  OR stretch muscle*  OR stretching muscle*  OR stretching-shortening cycle*  OR stretching exercise*  OR muscle stretching exercises*  OR muscle strain*  OR strained muscle*  OR stretch injured*  OR dynamic force spectroscopy*  OR stretch injury*  OR passive stretch*  OR active stretch* OR injury, repetition strain*  OR occupational overuse syndrome*  OR overuse syndrome*  OR repetition strain injury*  OR repetitive motion disorder*  OR repetitive strain injury*  OR strain injury, repetition*  OR repetitive stretch* OR mobilization* OR manual therapy* OR massage* OR eccentric muscle contraction* OR delayed onset muscle soreness*) *AND* **TOPIC:** (inflammation*  OR acute inflammation*  OR bacterial inflammation*  OR inflammation reaction*  OR inflammation response*  OR inflammatory condition*  OR inflammatory lesion*  OR inflammatory process*  OR inflammatory reaction*  OR inflammatory response*  OR inflammatory syndrome*  OR reaction, inflammation*  OR response, inflammatory*  OR serositis*  OR sterile inflammation*  OR inflammatory mediator*  OR specialized proresolving mediator*  OR inflammation resolution*) *NOT* **TOPIC:** (clinical trial*  OR Human* OR human trial* OR vascular*  OR lung*  OR respiratory*  OR reproductive* OR systematic review* OR meta-analysis* OR Pulmonary*  OR  Neural*  OR endothelial*  OR liver*  OR domestic animal*  OR intestinal*  OR brain*  OR heart*)  **Timespan:** 1900 – 2020. **Indexes:** SCI-EXPANDED, SSCI, A&HCI, CPCI-S, CPCI-SSH, BKCI-S, BKCI-SSH, ESCI, CCR-EXPANDED, IC. Quick filters: document type (articles); Languages: English. | **454** |
