# Supplementary material for: A systematic review of in vivo stretching regimens on inflammation and its relevance to translational yoga research
Source: PLoS One. 2022 Jun 1;17(6):e0269300. doi: 10.1371/journal.pone.0269300 (PMC9159623; doi:10.1371/journal.pone.0269300)
Supplement: S1 Protocol — (DOCX) [file pone.0269300.s002.docx]

| 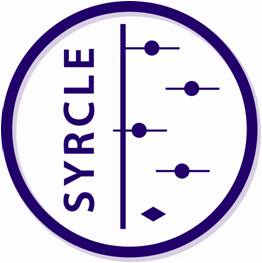 **Systematic Review Protocol for Animal Intervention Studies**  **Format by SYRCLE (**[**www.syrcle.nl**](http://www.syrcle.nl)**)**  **Version 2.0 (December 2014)** | | | | |
| --- | --- | --- | --- | --- |
| **Item #** | **Section/Subsection/Item** | **Description** | | **Check for approval** |
|  | A. General | | | |
| 1. | Title of the review | A systematic review of *in vivo* stretching regimens on inflammation and its relevance to translational yoga research | |  |
| 2. | Authors (names, affiliations, contributions) | Dennis Muñoz-Vergara. Division of Preventive Medicine (DPM), Brigham and Women’s Hospital (BWH), Harvard Medical School (HMS). Osher Center for Integrative Medicine (OCIM), Boston, MA, USA. Conceptualization, Data curation, Formal analysis, Funding acquisition, Methodology, Project administration, Writing – original draft, Writing – review & editing  Weronika Grabowska. DPM, BWH, HMS, Boston, MA, USA. Data curation, Formal analysis  Gloria Y. Yeh. Division of General Medicine and Primary Care, Beth Israel Deaconess Medical Center (BIDMC), HMS, Boston, MA, USA. Methodology, Supervision  Sat Bir Khalsa. DPM, BWH, HMS. OCIM, Boston, MA, USA. Validation, Writing – review & editing  Kristin L. Schreiber. Department of Anesthesiology, Perioperative and Pain Medicine, BWH, HMS, Boston, MA, USA. Validation, Writing – review & editing  Christene A. Huang. The Department of Surgery, School of Medicine, University of Colorado, Denver, CO, USA. Validation, Writing – review & editing  Ann Marie Zavacki. Division of Endocrinology, Diabetes, and Hypertension, BWH, HMS, Boston, MA, USA. Validation, Writing – review & editing  Peter M. Wayne. DPM, BWH, HMS, OCIM, Boston, MA, USA. Conceptualization, Funding acquisition, Methodology, Supervision, Writing – review & editing | |  |
| 3. | Other contributors (names, affiliations, contributions) | NA | |  |
| 4. | Contact person + e-mail address | Dennis Muñoz-Vergara:  [dmunozvergara@bwh.harvard.edu](mailto:dmunozvergara@bwh.harvard.edu) | |  |
| 5. | Funding sources/sponsors | T32AT00051— <https://www.nccih.nih.gov>  K24AT009282— <https://www.nccih.nih.gov>  R01DK044128— <https://www.niddk.nih.gov> K24AT009465— <https://www.nccih.nih.gov> | |  |
| 6. | Conflicts of interest | No conflicts of interests | |  |
| 7. | Date and location of protocol registration | - | |  |
| 8. | Registration number (if applicable) | - | |  |
| 9. | Stage of review at time of registration | - | |  |
|  | B. Objectives | | | |
|  | Background | | | |
| 10. | What is already known about this disease/model/intervention? Why is it important to do this review? | **Body stretching is an integral therapeutic component of mind-body exercises such as** yoga. Currently, few experimental studies have tried to isolate the impact of stretching from other potentially therapeutic **components (e.g., focused attention, breathing, psychosocial support).** Multifactorial additive designs and/or dismantling studies represent possible experimental clinical approaches for isolating and mechanistically evaluating the therapeutic impact of stretching. However, these approaches can be costly and premature. One strategy to inform future mechanistic clinical research design is to leverage *in vivo* rodents studies on the effect of stretching intervention targeting the musculoskeletal (MSK) and integumentary systems, both actively involved during yogic exercise, and summarize their findings on inflammatory outcomes | |  |
|  | Research question | | | |
| 11. | Specify the disease/health problem of interest | Activation/suppression of inflammatory processes due to MSK and integumentary systems stretching interventions using in vivo rodents models | |  |
| 12. | Specify the population/species studied | Laboratory rodents | |  |
| 13. | Specify the intervention/exposure | Active and passive stretching interventions of the MSK or integumentary systems | |  |
| 14. | Specify the control population | Independent non-stretched control group or the use of matched non-stretched contralateral body side or limb | |  |
| 15. | Specify the outcome measures | Inflammation-related outcomes included a broad array of measures such as mi­croscopic (e.g., myofiber degeneration), genetic (e.g., gene expression), cell/particle-sorting (e.g., cytokines), enzymatic (e.g., ROS), and macroscopic techniques (e.g., muscle or connective tissue ultrasound) | |  |
| 16. | State your research question (based on items 11-15) | Can pre-clinical (i.e., in vivo rodent models) studies employing active or passive stretching techniques of the MSK and integumentary systems help to understand yogic stretching and its impact on inflammation? | |  |
|  | C. Methods | | | |
|  | Search and study identification | | | |
| 17. | Identify literature databases to search (*e.g.* Pubmed, Embase, Web of science) | **X** MEDLINE via PubMed  **X** Web of Science  □SCOPUS **X** EMBASE  □Other, namely:    **X** Specific journal(s), namely: Harvard University library search platform (Hollis) | |  |
| 18. | Define electronic search strategies (*e.g.* use the [step by step search guide^15^](http://www.ncbi.nlm.nih.gov/pmc/articles/PMC3265183/pdf/LA-11-087.pdf) and animal search filters[^20,^](http://www.ncbi.nlm.nih.gov/pmc/articles/PMC3104815/pdf/LA-09-117.pdf) [^21^](http://lan.sagepub.com/content/48/1/88.full.pdf+html)) | Supplementary file containing search strategy:  "S1_Table.docx" | |  |
| 19. | Identify other sources for study identification | □Reference lists of included studies □Books  □Reference lists of relevant reviews  □Conference proceedings, namely:  □Contacting authors/ organisations, namely:  □Other, namely: | |  |
| 20. | Define search strategy for these other sources | - | |  |
|  | Study selection | | | |
| 21. | Define screening phases (*e.g.* pre-screening based on title/abstract, full text screening, both) | The Covidence^©^ software will be used for:  Phase 1: duplicate exclusion  Phase 2: screening based on title and abstract  Phase 3: full-text screening of the eligible articles | |  |
| 22. | Specify (a) the number of reviewers per screening phase and (b) how discrepancies will be resolved | Phase 1: will be done by Covidence^©^ software automatically  Phase 2: DMV and WG  Phase 3: DMV and WG  Differences will be solved through discussion or by consulting a third investigator (PW and GY) | |  |
|  | *Define all inclusion and exclusion criteria based on:* | | | |
| 23. | Type of study (design) | Inclusion criteria: comparison of the effect of different stretching techniques administered to the MSK or integumentary systems, either as an injurious or therapeutic intervention, and evaluating at least one outcome related to inflammation. Also, studies will require to include an independent non-stretched control group or the use of matched non-stretched contralateral body side or limb.  Exclusion criteria include rodent studies stretching other body systems (e.g., reproductive or respiratory systems), *in vitro* and *ex vivo* paradigms, and clinical trials | |  |
| 24. | Type of animals/population (*e.g.* age, gender, disease model) | Inclusion criteria: Healthy populations of rodents (i.e., rats and mice) of any gender or age will be considered | |  |
| 25. | Type of intervention (*e.g.* dosage, timing, frequency) | Inclusion criteria: passive or active stretching, injurious or therapeutic intent of stretching, single or multiple bouts of stretching, and short- or long-term stretching interventions | |  |
| 26. | Outcome measures | Inclusion criteria: Inflammation-related outcomes included a broad array of measures such as mi­croscopic (e.g., myofiber degeneration), genetic (e.g., gene expression), cell/particle-sorting (e.g., cytokines), enzymatic (e.g., ROS), and macroscopic techniques (e.g., connective tissue ultrasound).  Exclusion criteria: no relevant outcome measures | |  |
| 27. | Language restrictions | Inclusion criteria: English only  Exclusion criteria: any other non-English language | |  |
| 28. | Publication date restrictions | Inclusion criteria: 1900-2020  Exclusion criteria: Pre-1900 articles will be excluded because the laboratory techniques to evaluate inflammatory outcomes were not yet fully developed | |  |
| 29. | Other | NA | |  |
| 30. | Sort and prioritize your exclusion criteria per selection phase | Selection phase: duplicates removal by Covidence^©^ software  Selection phase: Title and abstract screening and full-text screening  1. Wrong intervention (e.g., no stretching intervention or no stretching of the MSK or integumentary systems)Other reviews  2. Wrong specie  3. Wrong outcome | |  |
|  | Study characteristics to be extracted (for assessment of external validity, reporting quality) | | | |
| 31. | Study ID (*e.g.* authors, year) | Authors, title, year. | |  |
| 32. | Study design characteristics (*e.g.* experimental groups, number of animals) | Randomization, number of rodents per batch/group, number of groups, presence of control group | |  |
| 33. | Animal model characteristics (*e.g.* species, gender, disease induction) | Strain, sex, rodent specie, age, N Rats and mice | |  |
| 34. | Intervention characteristics (*e.g.* intervention, timing, duration) | Type and intention of stretching: passive and/or active stretching. Injurious and/or therapeutic stretching. Pro- or anti-inflammatory study  Intervention name and protocol’s length (shorter vs. longer term protocols)  Stretching parameters: intensity, duration, and frequency | |  |
| 35. | Outcome measures | Inflammatory outcomes measured with different laboratory techniques, such as: immunohistochemistry (IHC); immunofluorescence (IF); quantitative reverse transcription polymerase chain reaction(RT-qPCR); ultrasound (US); Enzyme-Linked ImmunoSorbent Assay (ELISA); terminal deoxynucleotidyl transferase dUTP nick end labeling (TUNEL); western blot (WB); RNA sequencing (RNA-seq); second harmonic generation microscopy (SHG); flow cytometry (FC); Lipidomics (SPMs, specialized pro-resolving mediators); *in vivo* bioluminescent imaging (*In vivo* BI)  Narrative results of inflammatory outcomes | |  |
| 36. | Other (*e.g.* drop-outs) | - | |  |
|  | Assessment risk of bias (internal validity) or study quality | | | |
| 37. | Specify (a) the number of reviewers assessing the risk of bias/study quality in each study and (b) how discrepancies will be resolved | a) 2 reviewers. The criteria will be independently assessed by DMV and WG by using collectively predefined assessment criteria. b) discrepancies will be resolved by discussion | |  |
| 38. | Define criteria to assess (a) the internal validity of included studies (*e.g.* selection, performance, detection and attrition bias) and/or (b) other study quality measures (*e.g.* reporting quality, power) | X By use of [SYRCLE's Risk of Bias tool^4^](http://www.biomedcentral.com/1471-2288/14/43/abstract)  □By use of SYRCLE’s Risk of Bias tool, adapted as follows:  □By use of [CAMARADES' study quality checklist, e.g ^22^](http://www.ncbi.nlm.nih.gov/pubmed/15060322)  □Other criteria, namely:. | |  |
|  | Collection of outcome data | | | |
| 39. | For each outcome measure, define the type of data to be extracted (*e.g.* continuous/dichotomous, unit of measurement) | Narrative results of inflammatory outcomes | |  |
| 40. | Methods for data extraction/retrieval (*e.g.* first extraction from graphs using a digital screen ruler, then contacting authors) | Extraction from text and tables | |  |
| 41. | Specify (a) the number of reviewers extracting data and (b) how discrepancies will be resolved | a) Two reviewers (DMV and WG) will extract all data. b) discrepancies will be resolved by discussion and if an agreement is not reached, discrepancies will be discussed by two additional independent reviewers (PW and GY) | |  |
|  | Data analysis/synthesis | | | |
| 42. | Specify (per outcome measure) how you are planning to combine/compare the data (*e.g.* descriptive summary, meta-analysis) | -Descriptive summary alone | |  |
| 43. | Specify (per outcome measure) how it will be decided whether a meta-analysis will be performed |  | |  |
|  | *If a meta-analysis seems feasible/sensible, specify (for each outcome measure):* | | | |
| 44. | The effect measure to be used (*e.g.* mean difference, standardized mean difference, risk ratio, odds ratio) | - | |  |
| 45. | The statistical model of analysis (*e.g.* random or fixed effects model) | - | |  |
| 46. | The statistical methods to assess heterogeneity (*e.g.* I^2^, Q) | - | |  |
| 47. | Which study characteristics will be examined as potential source of heterogeneity (subgroup analysis) | - | |  |
| 48. | Any sensitivity analyses you propose to perform | - | |  |
| 49. | Other details meta-analysis (*e.g.* correction for multiple testing, correction for multiple use of control group) | - | |  |
| 50. | The method for assessment of publication bias | - | |  |
|  | | | | |
| Final approval by (names, affiliations): | |  | Date: | |
